# Investigating Children’s Exposure to Outdoor Food Marketing in 2 Swedish Cities Using a Smartphone App: Cross-Sectional Study

**DOI:** 10.2196/70192

**Published:** 2026-03-24

**Authors:** Sofia Spolander, Petter Fagerberg, Billy Langlet, Ioannis Sarafis, Ioannis Ioakimidis

**Affiliations:** 1 Department of Medicine, Huddinge, Karolinska Institutet, Blickagången 16, Stockholm, 14152 Flemingsberg, Sweden, 46 736508228; 2 Karolinska Institutet, Stockholm, Sweden; 3 Department of Electrical and Computer Engineering, Aristotle University of Thessaloniki, Thessaloniki, Greece

**Keywords:** food environment, marketing, advertisement, ultraprocessed food, unhealthy foods, children, adolescents, obesity, social class, children-centric approach

## Abstract

**Background:**

Childhood obesity and unhealthy dietary habits remain major public health concerns and are influenced by the surrounding food environment. Food marketing, particularly for ultraprocessed foods (UPFs), shapes children’s food preferences and consumption. However, food environments are complex and constantly changing, making them difficult to map and monitor. Developing approaches that capture these dynamics is essential to understand and address children’s exposure to unhealthy food marketing.

**Objective:**

This study aimed to pilot-test a novel tool consisting of a smartphone app and dashboard designed to identify areas where children are exposed to outdoor food advertising. Additionally, the study assessed the prevalence of advertisements for UPFs, health-promoting foods, and offers in the identified areas and explored differences in exposure by city size and socioeconomic status (SES).

**Methods:**

A cross-sectional study was performed in 2 Swedish counties. Initially, 46 children from 4 schools in areas with varying SES used a smartphone app to take pictures of food advertisements that they encountered in their everyday lives. The app also recorded the GPS locations of where the pictures were taken. Pictures with associated GPS data were automatically uploaded and visualized in a web-based dashboard, allowing for identification of areas where children see many food advertisements, so-called “hotspot areas.” The identified hotspot areas were subsequently visited by 2 researchers (SS and PF), who systematically photographed all food advertisements in the areas. All pictures taken by the researchers were later analyzed based on their content of UPFs, health-promoting foods such as fruits, berries, vegetables, and seafood (FBVS), and price promotions.

**Results:**

Based on 1308 pictures of outdoor food advertisements taken by children using the app, 34 hotspot areas were identified through the dashboard. In these areas, researchers collected 2955 pictures of outdoor food advertisements during the mapping activity. Overall, 77.5% (2291/2955) of advertisements promoted UPFs, with no significant difference between the large and small cities. In Stockholm, a higher proportion of UPFs appeared in the high-SES area compared to the low-SES area, though the proportion was high in both areas. FBVS featured in 20.8% (616/2955) of advertisements, slightly more often in Stockholm and in the high-SES area. Price promotions appeared in 23.6% (697/2955) of advertisements, mainly featured UPFs (518/697, 74.3%) and less often FBVS (142/697, 20.4%). Price promotions for UPFs were somewhat more frequent in Stockholm, and FBVS promotions were more common in the high-SES area.

**Conclusions:**

Using a novel, child-centered, mobile health tool combining a smartphone app and dashboard, this study identified local food advertising hotspots. Most advertisements in the hotspots promoted UPFs, while a few featured FBVS, a pattern also reflected in price promotions. These trends were consistent across areas, highlighting a food marketing landscape misaligned with dietary guidelines and the need for policy action.

## Introduction

The prevalence of childhood obesity has increased dramatically in Sweden over the past few decades, following global trends [1,2]. This is concerning since childhood obesity is associated with an increased risk of several chronic diseases, including type 2 diabetes and cardiovascular disease, as well as various psychological problems, including low self-esteem and depression [3,4]. Moreover, childhood obesity increases the risk of obesity and developing chronic diseases in adulthood [5,6].

Poor dietary habits are a major modifiable risk factor for obesity. In recent years, increasing attention has been given to the role of the food environment in shaping poor dietary habits [7]. In parallel with the increase in obesity, the food environment has undergone rapid and widespread changes, resulting in increased access to cheap foods that are low in nutrients and high in calories, such as ultraprocessed foods (UPFs) [8]. High availability and consumption of UPFs have been associated with increased risk of obesity in both children [9] and adults [10,11], as well as an increased risk of all-cause mortality [12,13]. Conversely, the consumption of foods such as fruits, vegetables, whole grains, fish, and seafood is associated with protective effects against obesity and noncommunicable diseases [14]. This contrast points to the value of mapping food environments to inform strategies that promote healthier diets.

Food marketing represents a key element of the food environment, influencing food choices and consumption patterns. A growing number of studies show that the physical food marketing landscape is dominated by advertisements for foods associated with negative health outcomes such as UPFs [15]. A review assessing studies from 21 countries found that on average, 63% of food advertisements promoted unhealthy foods [15]. However, different studies use different terminology, including UPFs, “discretionary foods,” “noncore foods,” and “junk food,” to describe more or less the same type of unhealthy food items [15]. Studies investigating food advertisements around schools have found that the vast majority (~60%-90%) of food advertisements market unhealthy foods [16-23].

Food environments dominated by UPF marketing pose a health risk, as food marketing has been linked to increased intake, altered preferences, and purchase requests in children [24]. Experimental studies show that children exposed to food advertisements, on television or in advergames, consume more calories than those exposed to nonfood advertisements [25-27]. These effects are greater in children with overweight or obesity [25], and exposure increases the likelihood of choosing advertised products [27]. The impact of promoting healthier foods remains uncertain, as most studied products are of low nutritional value [27].

Beyond general exposure, the distribution of food advertising may also vary by socioeconomic status (SES). Our previous study conducted in 2 Stockholm neighborhoods, 1 with low SES with higher rates of childhood obesity and 1 with high SES with lower rates of childhood obesity, found that 65% of food advertisements promoted UPFs, with a higher proportion in the low-SES area (73%) compared to the high-SES area (59%) [28]. Studies from other countries have shown mixed results in regard to SES differences [15].

Mapping and measuring food environments, including exposure to food advertising, present several challenges due to their constant change and complexity [29]. This underscores the need for methods that enable continuous or repeated assessments without requiring extensive resources. To fully understand the scope and public health implications of food environments, it is also essential to use diverse measurement approaches that address multiple dimensions of the food environment [29]. Mobile health (mHealth) technologies, such as smartphone apps, offer a promising solution by facilitating real-time data collection directly from participants. These tools can capture locational, behavioral, and environmental information, allowing researchers to identify patterns of exposure and behavior in different groups or individuals and over time. Integrating mHealth approaches can therefore enhance both the efficiency and depth of environmental assessment, providing valuable insights into how food marketing and other environmental factors influence health outcomes and inform policy design.

While previous studies have investigated the outdoor food advertising landscape around schools and other locations frequently visited by children, the mapped areas have typically been defined by researchers [16-23,28,30]. In contrast, this study adopts a child-centered approach to investigate children’s exposure to outdoor food advertising.

Given this context, the objectives of this study were (1) to pilot-test a novel tool, including a smartphone app and a dashboard, designed to identify areas where children are exposed to outdoor food advertising; (2) to assess the proportion of UPFs, health-promoting foods (such as fruits and vegetables), and price promotions for food and beverages within the identified areas; and (3) to examine potential differences in exposure by city size and SES.

## Methods

### Study Design

**Figure 1.**
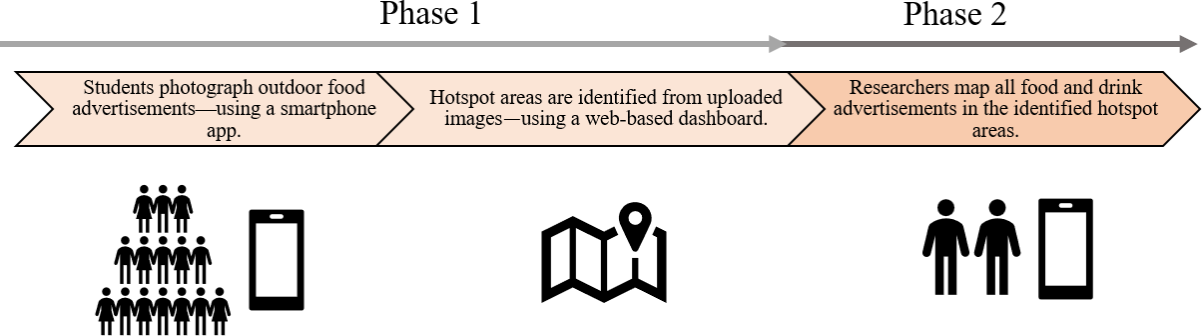
Schematic overview of the study design.

A cross-sectional study was performed from April to June 2022. The study took place in 2 Swedish counties: Stockholm County (Stockholm) with approximately 2.5 million inhabitants (in 2024) [31] and Gävleborg County (Gävleborg), located approximately 200 km north of Stockholm, with approximately 0.3 million inhabitants (in 2024) [31]. In phase 1 of the study, a tool consisting of a mobile app and a dashboard was used to identify areas where children are exposed to many food advertisements (hotspot areas). In phase 2, researchers systematically mapped all food advertisements within the identified hotspot areas to quantify the types of food being advertised (see Figure 1 for a schematic presentation of the study design).

### Participants and Recruitment

Children were recruited from secondary schools in Stockholm and Gävleborg. The aim was to include schools from areas with different SES in both a larger and a smaller city. Recruitment began by contacting schools in areas with varying SES levels via email and inviting them to participate. In total, 8 schools initially expressed interest, and online meetings were held to provide teachers with detailed information about the study. Ultimately, 4 schools were included based on expressed interest and scheduling availability during the study period.

In Stockholm, the capital of Sweden, areas with high and low SES were clearly distinguishable. In contrast, in Gävleborg, which is a smaller county, areas were more mixed. Consequently, clear SES differences were only present between the schools in Stockholm. The SES of the included areas was determined using the Segregation Barometer, a tool developed by the Swedish National Board of Housing, Building and Planning that incorporates statistics on education, income, health, and related factors [32].

The four participating schools were located (1) in Östermalm, an urban area in central Stockholm with high SES (~83,000 inhabitants in 2021); (2) in Södertälje, an urban area in the outskirts of Stockholm with low SES (~76,000 inhabitants in 2020); (3) in central Gävle, an urban area with mixed SES; and (4) in Bomhus, an area with mixed SES in the outskirts of Gävle (~79,000 inhabitants in Gävle in 2020). Each of the 4 participating schools selected 1 or 2 school classes with students aged 13 to 14 years for participation in the study. Given the exploratory nature of the study, the sample size was determined based on feasibility and participant accessibility, rather than formal statistical calculations.

### Data Collection Tools: Mobile App and Dashboard

**Figure 2.**
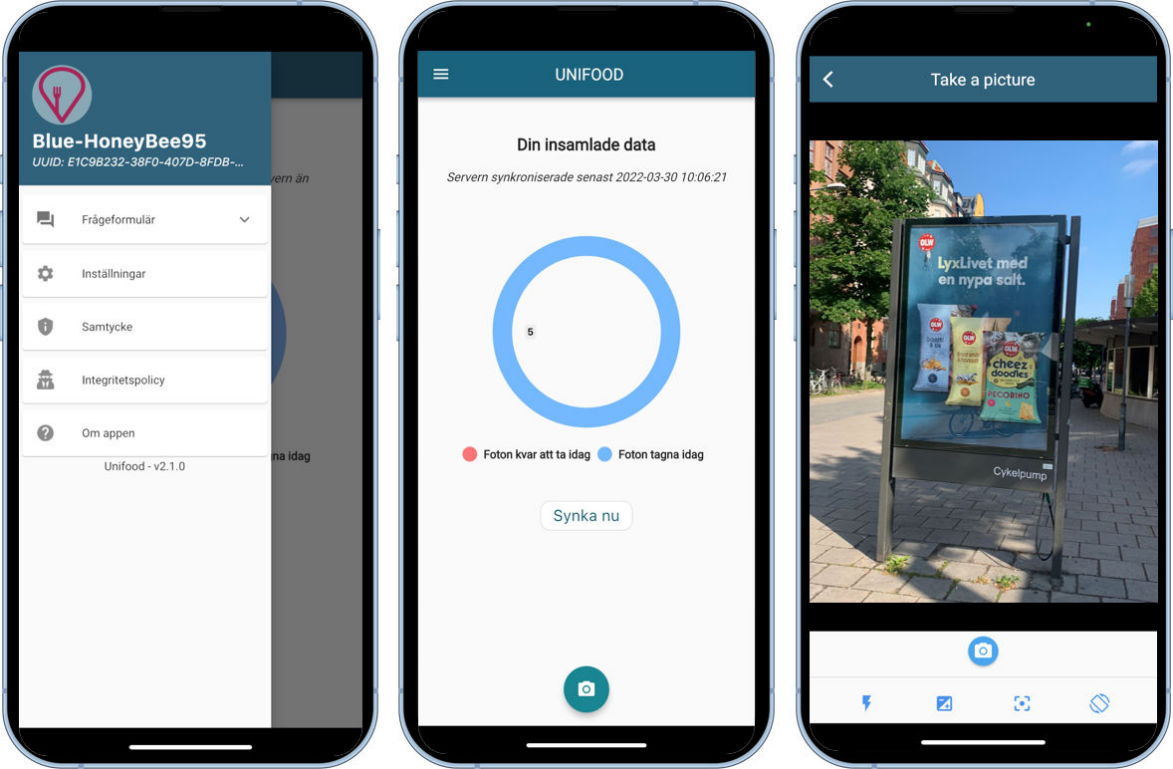
The mobile app used by the participants to take pictures of food advertisements. Left: the menu with information about the username and access to questionnaires and settings. Middle: the main screen of the smartphone app illustrating the daily picture contribution in blue. Right: the camera function.

The data collection tools for this study consisted of a custom-developed smartphone app and a purpose-built web-based dashboard. These tools were designed primarily to facilitate the identification of hotspot areas based on participant reports. The app’s key feature was a camera function that allowed users to take pictures of outdoor food advertisements (Figure 2). Along with the picture itself, the app recorded the GPS location of where it was taken. When the smartphone was connected to the internet (via Wi-Fi or mobile data), the pictures with associated GPS data were automatically uploaded to a secure server and visualized in the dashboard. The app also included a function that assigned users random usernames upon registration in the app, a questionnaire in which users could answer questions about their gender, age, height, and weight (Figure 2), and visual feedback on each user’s daily photo contributions (Figure 2).

**Figure 3.**
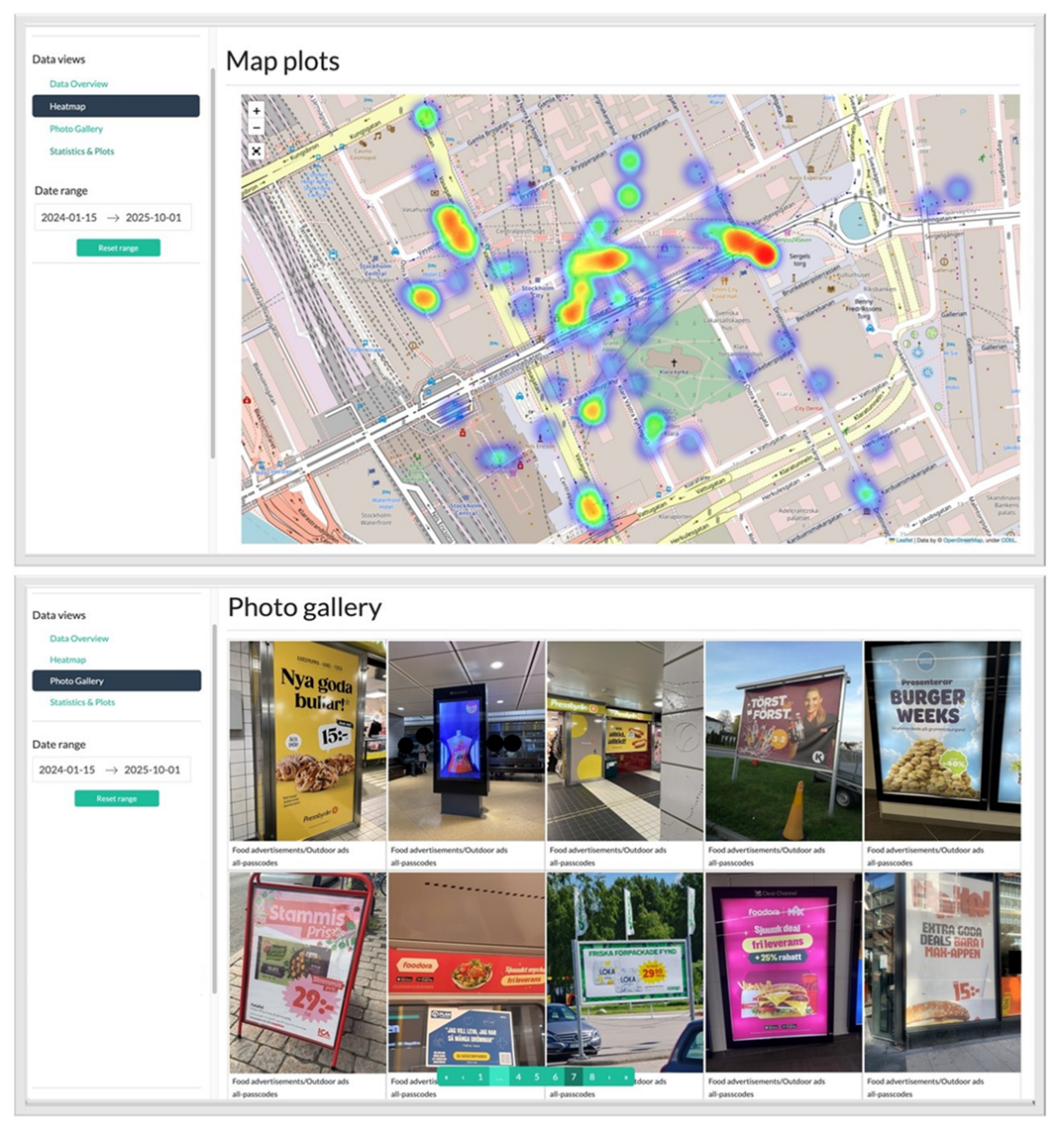
The dashboard. Top: heat map visualization used to identify hotspot areas. Bottom: picture gallery.

The dashboard (Figure 3) was a cloud-based web service accessible through a web browser. It was developed in Python (Python Software Foundation) using Dash and the Flask web framework. Heat map visualizations were implemented using the Leaflet library. Its main purpose was to provide heat maps of aggregated GPS-tagged data collected from the app along with basic metadata filtering functionalities (eg, date range and photo category). Additionally, it included a photo gallery for quick inspection of the collected data, as well as simple statistical plots and visualizations. The app and dashboard were developed by the Multimedia Understanding Group, Department of Electrical and Computer Engineering, Aristotle University of Thessaloniki, Greece.

### Phase 1: Identification of Hotspot Areas Using the App and Dashboard

**Figure 4.**
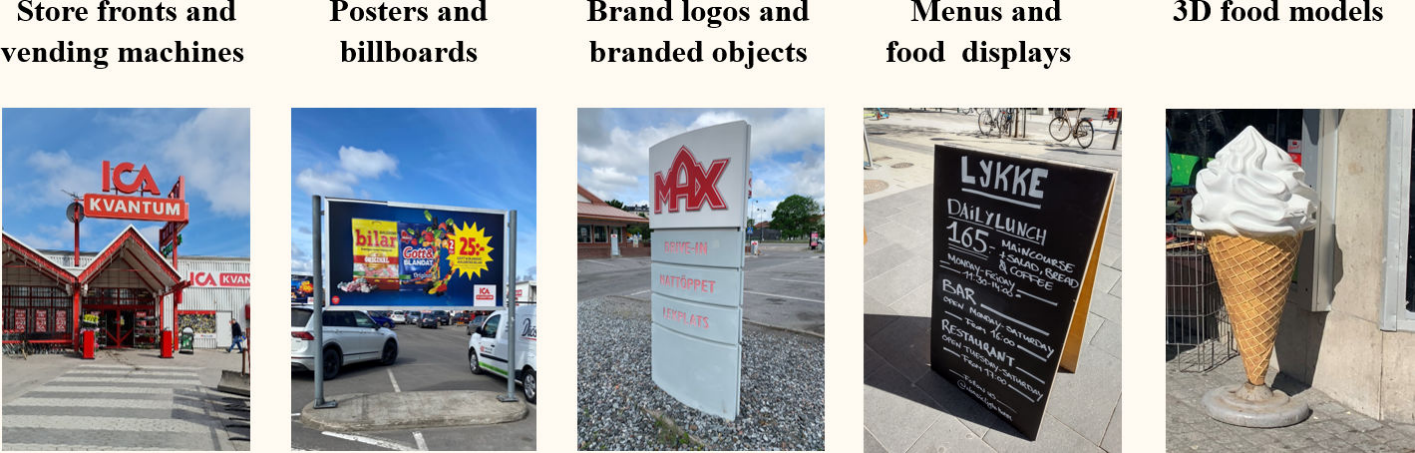
Different types of physical food advertisements common in the Swedish context that were considered in the study.

The study started with a session, during which researchers helped the participating children to install the app on their own smartphones. After downloading the app, children registered and answered the in-app questionnaire. Participants then received information from the researchers on how to use the app, including how to take pictures of outdoor food advertisements. They were instructed to avoid taking pictures where people’s faces were visible and to avoid taking pictures at their home address. Moreover, participants were informed that the study duration was 2 weeks and that they should take a minimum of 5 pictures of outdoor food advertisements per day, but only the ones that they encounter while living their normal lives (ie, not go out of their way to find food advertisements). Participants were instructed that they could photograph the same advertisement multiple times if they encountered it on different occasions (eg, seeing it daily on their way to school), but only once per occasion. Finally, participants were instructed on what constitutes an outdoor food advertisement with examples (Figure 4). Advertisements in public transportation systems, such as in the subway or on buses, as well as general spaces in shopping malls, were considered as outdoor advertisements, whereas in-store advertisements were not.

**Figure 5.**
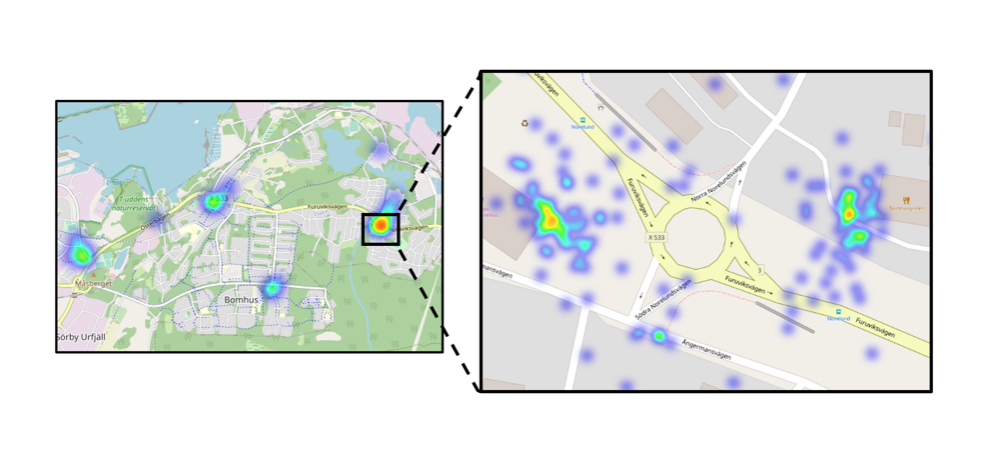
Illustration of the heat maps used to identify hotspot areas at 2 different zoom levels (displayed in the web-based dashboard).

After 2 weeks of data collection, areas where the children reported many food advertisements, so-called “hotspot areas,” could be identified via the web-based dashboard (Figure 5). An area was classified as a hotspot if more than 5 pictures of food advertisements were located within a ~50 m radius.

### Phase 2: Complementary Researcher Data Collection

Prior to mapping all food advertisements within the identified hotspot areas, the researchers conducted a training session to ensure consistency and reduce the risk of observer bias. During the training session, the 2 researchers independently mapped a nonhotspot area to ensure a similar approach to mapping of advertisements. Any differences in mapping strategies and definitions were discussed, resulting in a more clearly defined food advertisement mapping protocol.

After the training session, each hotspot area identified through the dashboard was converted to a square-shaped hotspot buffer, measuring either 100×100 m or 250×250 m, depending on the number and spatial distribution of pictures taken by children in that area. These buffers served as guides for researchers, indicating where to take pictures of food advertisements. Between June 7 and June 14, 2022, a total of 2 researchers systematically visited each buffer and photographed every food advertisement in all hotspot buffers using the app.

### Picture Annotations

An annotation scheme previously developed by the research group [28] was adapted for this study and used as a guideline for annotating the collected pictures. All pictures of food advertisements taken by the researchers in the hotspot areas were annotated based on the content of the following foods: UPFs (including pizza, burgers, kebab, sweet snacks, salty snacks, readymade meals, sugary cereal, sweetened dairy products, sugary drinks, and artificially sweetened beverages; a full list of food and beverages included in the category is available in Multimedia Appendix 1), fruits, berries, and/or vegetables, and fish and/or other seafood. If an advertisement illustrated a meal, more than one-third of the meal had to contain fruits, berries, or vegetables to be annotated as containing fruits, berries, or vegetables (eg, salad on a hamburger or a berry on top of a dessert did not meet the criteria). Logos of well-known fast-food restaurants were annotated as fast food, whereas logos of grocery stores were not assigned to any specific food category. A single image could include multiple categories, for example, both UPFs and fruits, berries, or vegetables.

Two trained nutritionists (PF and SS) annotated all pictures independently using the VIA annotation software for images, audio, and video (Visual Geometry Group, University of Oxford). The pictures were presented in a random order to reduce the risk of location-related bias. Annotation disagreements were discussed until consensus was reached between the 2 researchers. Following this process, the categories fruits, vegetables, and/or berries and fish and other seafood were combined into the composite category “fruits, vegetables, berries, and seafood” (FBVS). Subsequently, 1 trained nutritionist (SS) annotated all pictures to identify the presence of price promotions (eg, discounts, multibuy offers, or super-size deals).

### Statistical Analysis

All statistical tests were performed using R software (R Foundation for Statistical Computing). The hypothesis and analysis methods were specified before data collection.

The Pearson chi-square test was selected as the analysis method to compare the proportion of different food advertisements, due to all cells having more than 5 observations. The chi-square tests were performed to compare the proportions of advertisements containing UPFs, FBVS, and price promotions between the included schools based on (1) in what city the school was located (larger vs smaller city) and (2) the SES of the area in which the school was located (low vs high SES). For the comparison based on city size, the 2 schools in Gävleborg were compared against the 2 schools in Stockholm. For the comparison based on SES, only the 2 schools in Stockholm were compared, since there was no clear difference in SES between the 2 areas in Gävleborg.

To evaluate interrater agreement between the 2 researchers’ annotations, Cohen κ was used. κ value of <0.21 was interpreted as “no agreement,” 0.21‐0.39 “minimal agreement,” 0.40‐0.59 “weak agreement,” 0.60‐0.79 “moderate agreement,” 0.80‐0.90 “strong agreement,” and >0.90 as “almost perfect agreement” [33]. The interrater analysis was performed prior to the consensus discussion between the 2 researchers.

### Ethical Considerations

This study was approved by the Swedish Ethical Review Authority (DNR 2021-06800-01). Prior to participation, the researchers visited children in their classrooms to provide them with detailed information about the study, including what it means to participate, that participation is voluntary, and that they are free to withdraw consent at any time. If a participant chose to withdraw their consent, they could also request the deletion of all their collected data. Children were also provided with information letters with consent forms for both themselves and their legal guardians to sign if they agreed to participate. Other than providing signed consent, there were no additional inclusion or exclusion criteria. As compensation, individuals received 1 cinema ticket for their participation, regardless of the number of pictures submitted, once the data collection period was completed. To ensure participant privacy, all collected data were pseudonymized. Upon registration in the app, each participant was automatically assigned a random username, which was linked to their uploaded data within the data collection system. As a result, the data remained fully anonymous within the system. However, a separate key file containing the link between usernames and participants’ names and schools was securely stored outside the data collection system. This file was accessible only to the research team and was used exclusively to identify users who might have uploaded inappropriate content, such as images containing identifiable personal information, if necessary. All uploaded data were stored on secure servers, accessible only by selected members of the research team. Submitted pictures were reviewed, and pictures not meeting the study’s criteria (eg, those unrelated to outdoor food or beverage advertisements) were permanently deleted.

## Results

### Participant Characteristics (Phase 1)

A total of 46 children participated in the study, and they collected a total of 1308 pictures of food and beverage advertisements using the app. The participant characteristics and the number of pictures taken by the participants in different areas can be seen in Table 1.

**Table 1.** Participant characteristics and number of pictures taken by the participants in Stockholm and Gävleborg.

School	Participants, n (%)	Sex		Age^a^ (years), mean (SD)	BMI^b^ (kg/m^2^), mean (SD)	Total pictures taken^c^, n (%)[Table-fn T1_FN3]	Relevant pictures, n (% of all pictures)	Total pictures per participant, mean (SD)
		Female, n (%)	Male, n (%)					
Stockholm								
Östermalm	15 (32.6)	11 (73)	4 (27)	14.2 (0.4)	18.1 (2.0)	487 (36.7)	485 (99.5)	32.5 (33.8)
Södertälje	7 (15.2)	3 (43)	4 (57)	13.9 (0.3)	19.7 (1.9)	206 (15.5)	204 (99)	29.4 (14.4)
Total Stockholm	22 (47.8)	14 (54)	8 (46)	14.1 (0.4)	18.7 (2.1)	693 (52.2)	689 (99.4)	31.5 (28.7)
Gävleborg								
Central Gävle	5 (10.9)	0 (0)	5 (100)	14.8 (0.2)	19.8 (1.6)	147 (11.1)	144 (98)	29.4 (35.4)
Bomhus	19 (41.3)	5 (26)	14 (74)	13.8 (0.4)	20.6 (3.3)	488 (36.7)	475 (97.3)	25.7 (19.6)
Total Gävleborg	24 (52.2)	5 (21.0)	19 (79)	14.0 (0.5)	20.5 (3.1)	635 (47.8)	619 (97.5)	26.5 (22.8)
Total	46 (100)	19 (41)	27 (59)	14.0 (0.5)	19.7 (2.8)	1328 (100)	1308 (98.6)	28.9 (25.6)

a Data missing for 3 participants in Östermalm and 1 participant in central Gävle.

b Data missing for 4 participants in Östermalm, 1 participant in Södertälje, and 1 participant in central Gävle.

c Including pictures that were not in the scope of the study.

### Characteristics of the Identified Hotspot Areas (Phase 1)

**Figure 6.**
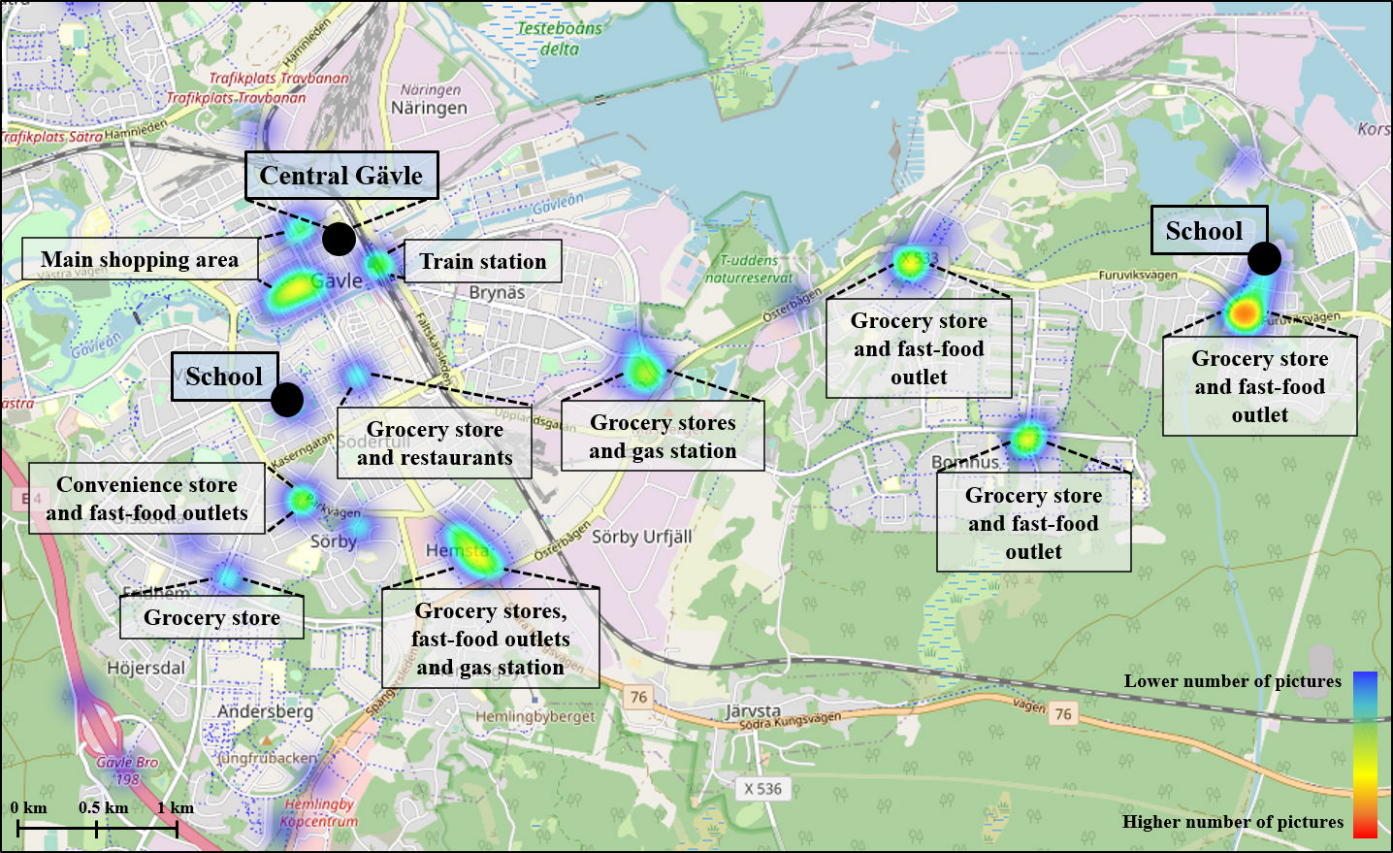
Map depicting the identified hotspot areas in Gävleborg, along with the types of establishments located within each area. The map was extracted from the research dashboard, with labels manually added to indicate the specific establishments present in the areas. The color gradient reflects the number of images associated with each hotspot, ranging from dark blue (indicating a low number of images) to red (indicating a high number of images).

Across the 2 counties, 34 hotspot areas were identified using the tool, covering a total area of 740,000 m^2^ (74 hectares). At least 1 hotspot area was identified near each school. These areas typically included local grocery stores, convenience stores, and/or fast-food outlets. In Gävleborg and Södertälje, additional hotspot areas were identified further from the schools, also containing similar types of food establishments. Both locations also had hotspot areas located in larger shopping areas. In central Stockholm (Östermalm school), hotspot areas were primarily concentrated in and around subway stations used by children, as well as in shopping malls and outdoor shopping areas. Figure 6 presents a map of the hotspot areas in Gävleborg extracted from the dashboard, along with examples of the types of establishments identified within these areas. Corresponding maps for Stockholm (Östermalm and Södertälje) are provided in Multimedia Appendix 2.

### Mapping of Hotspot Areas by the Researchers (Phase 2)

A total of 2955 pictures were taken during the food advertisement mapping activity conducted by the researchers. The number and total size of the identified hotspot areas as well as the number of pictures taken in the hotspot areas were greater in Stockholm compared to Gävleborg (Table 2). However, the mean number of pictures taken per hectare was similar in the 2 counties (42 pictures per hectare in Stockholm vs 37 pictures per hectare in Gävleborg).

**Table 2.** Presentation of the number and the size of the hotspot areas per city and school and the number of pictures taken by the researchers in each of the hotspot areas.

School	Hotspot areas, n/N (%)	Size (hectare)	Pictures, n/N (%)	Pictures per hectare
Stockholm	22/34 (64.7)	46.5	1948/2955 (65.9)	41.9
Östermalm	14/34 (41.2)	28.3	1350/2955 (45.7)	47.8
Södertälje	8/34 (23.5)	18.3	598/2955 (20.2)	32.8
Common for both schools	0/34 (0)	—^a^	—	—
Gävleborg	12/34 (35.3)	27.5	1007/2955 (34.1)	36.6
Central Gävle	2/34 (5.9)	2.0^b^	77/2955 (2.6)	38.5
Bomhus	5/34 (14.7)	8.0^b^	372/2955 (12.6)	46.5
Common for both schools	5/34 (14.7)	17.5	558/2955 (18.9)	31.9
Total	34/34 (100)	74.0	2955/2955 (100)	39.9

a Not applicable.

b Excluding areas common for both schools in Gävleborg.

### Proportions of Different Foods in Advertisements

#### UPFs

The analysis of the pictures of food advertisements taken by the researchers in all hotspot areas combined showed that 77.5% (2291/2955) of all food advertisements advertised UPFs. When comparing hotspot areas in Stockholm against hotspot areas in Gävleborg, no significant difference was found in proportion of UPFs being advertised (Stockholm: 1505/1948, 77.3% vs Gävleborg: 786/1007, 78.1%; *χ*^2^_1_=0.2; *P*=.62). However, a significant difference was observed in the proportion of UPFs advertised between the low- and high-SES areas in Stockholm, with a higher proportion in the high-SES area (Södertälje: 443/598, 74.1% vs Östermalm: 1063/1350, 78.7%; *χ*^2^_1_=5.0; *P*=.03).

#### Fruits, Vegetables, Berries, and Seafood

Overall, 20.8% (616/2955) of all food advertisements promoted FBVS. The proportion of FBVS advertisements was higher in Stockholm compared to in Gävleborg (Stockholm: 445/1948, 22.8% vs Gävleborg: 171/1007, 17%; *χ*^2^_1_=13.8; *P*<.001) and in the high-SES area in Stockholm compared to the low-SES area (Södertälje: 116/598, 19.4% vs Östermalm: 329/1350, 24.4%; *χ*^2^_1_=5.8; *P*=.02).

#### Price Promotions

Of all food advertisements in all areas combined, 23.6% (697/2955) included a price promotion. The proportion of advertisements including price promotions was slightly higher in Stockholm compared to Gävleborg (Stockholm: 490/1948, 25.2% vs Gävleborg: 207/1007, 20.6%; *χ*^2^_1_=7.8; *P*=.005). No difference in the proportion of price promotions was found when comparing the low- and high-SES areas in Stockholm (Södertälje: 164/598, 27.4% vs Östermalm: 326/1350, 24.1%; *χ*^2^_1_=2.4; *P*=.12).

Of all price promotions in all areas combined, 74.3% (518/697) advertised UPFs (compared to 1773/2258, 78.5% of advertisements without a price promotion;* χ*^2^_1_=5.4; *P*=.02). The proportion of price promotions that advertised UPFs was significantly higher in Stockholm compared to Gävleborg (Stockholm: 376/490, 76.7% vs Gävleborg: 142/207, 68.6%; *χ*^2^_1_=5.1; *P*=.03). There was no significant difference in the proportion of price promotions promoting UPFs when comparing the low- and high-SES areas of Stockholm (Södertälje: 128/164, 78% vs Östermalm: 248/326, 76.1%; *χ*^2^_1_=0.2; *P*=.63).

Moreover, of all price promotions in all areas combined, 20.4% (142/697) advertised FBVS (compared to 472/2258, 21% of advertisements without price promotions; *χ*^2^_1_=0.1; *P*=.73). There was a higher proportion of advertisements containing price promotions that advertised FBVS in Stockholm compared to in Gävleborg (Stockholm: 110/490, 22.4% vs Gävleborg: 32/207, 15.5%; *χ*^2^_1_=4.4; *P*=.04). A significant difference was also observed when comparing the proportion of price promotions advertising FBVS between the low- and high-SES areas of Stockholm, with a higher proportion found in the high-SES area (Södertälje: 23/164, 14% vs Östermalm: 87/326, 26.7%; *χ*^2^_1_=10.1; *P*=.002).

### Interrater Agreement

“Moderate agreement” (κ=0.762) was observed between the 2 raters for the categorization of advertisements advertising UPF out of total food advertisements. “Moderate agreement” was also observed for the categorization of fruits, berries, and/or vegetables (κ=0.720), as well as for the categorization of fish and other seafood out of total foods (κ=0.775).

## Discussion

### Principal Findings

This study used a novel mHealth tool, comprising an app and a dashboard, to identify hotspot areas where children are exposed to food advertisements. It also examined the content of food advertisements within the identified areas.

Using the tool, a total of 34 hotspot areas were identified. These included transportation hubs, shopping streets and malls, grocery stores, and fast-food restaurants, both in proximity to schools and further away. More than three-quarters of the food advertisements in the hotspot areas promoted UPFs, such as junk food, ready-to-eat meals, sweet and salty snacks, and sweetened drinks. Fewer than one-quarter of the advertisements advertised FBVS. Price promotions were present in nearly one-quarter of advertisements, predominantly targeting UPFs.

### Comparison With Prior Work

#### Data Collection and Analysis Methodology

Previous research has used child-centric approaches using mHealth technology to investigate children’s exposure to food advertising. For instance, the Kids’Cam study [34] combined GPS tracking and wearable cameras to objectively capture children’s exposure to food environments, providing detailed and comprehensive data on children’s exposure to food marketing. While such approaches generate rich information, they are resource-intensive and might raise privacy and data management challenges. This study offers an alternative approach to investigating children’s exposure to food advertising, using an app for child-led documentation that is both scalable and privacy-sensitive.

#### Analysis Outcomes

The dominance of food advertisements for UPFs aligns with findings from Sweden and other countries [15-20]. Given that children’s diets remain high in sugar, fat, and salt and low in whole grains, fruits, and vegetables, the current marketing landscape raises concerns [35]. Several countries have introduced policies to restrict unhealthy food marketing in public spaces to limit children’s exposure to marketing for unhealthy foods [36,37]. The results of this study suggest that similar measures may be relevant for Sweden.

A higher proportion of UPF advertisements was observed in the high-SES area of Stockholm compared to the low-SES area, contradicting findings from a previous study by our research group [28]. The earlier study mapped food advertisements in only 1 location per neighborhood (Skärholmen vs Östermalm) and found a higher proportion of UPF advertisements in the low-SES area. In contrast, this study included multiple locations within each area, offering a more comprehensive assessment of advertising exposure. Although the difference was statistically significant (1063/1350, 78.7% vs 443/598, 74.1%; *P*=.03), the high levels in both areas in this study suggest that the primary public health concern is the overall prevalence of UPF marketing, rather than SES-based differences.

Similar to previous studies [15,28], advertisements for health-promoting foods such as FBVS were scarce in the outdoor food advertising landscape. Other categories of health-promoting foods, such as products with whole grains or healthy fats, were not included in this study due to their extremely low prevalence (<1% of the advertisement dataset).

This study also explored price promotions, which are known to increase consumer purchases and are more frequently used for unhealthy foods compared to healthy foods [38]. While this is a recognized marketing strategy, few studies have examined its use in outdoor food advertising. One study conducted in the United States found that 19% of food advertisements around schools included price promotions but did not specify the types of foods promoted [39].

In our study, a significantly higher share of price promotions targeted UPFs in Stockholm compared to Gävleborg. This difference likely reflects the distinct characteristics of the hotspot areas. Hotspot areas in Stockholm often included subway stations with convenience stores, which frequently have price promotions for UPFs, while hotspot areas in Gävleborg more commonly featured grocery stores that generally have price promotions for a broader range of products. Moreover, within Stockholm, price promotions for fruits and vegetables were more prevalent in the high-SES area than in the low-SES area. Stockholm also had a higher overall proportion of price promotions for FBVS compared to Gävleborg. These patterns may reflect differences in consumer demand or retail marketing strategies across neighborhoods and could have potential public health implications. However, the limited number of participants submitting photos, especially in the low-SES area of Stockholm, may affect the representativeness of the identified hotspot areas, and the results should therefore be interpreted with caution.

### Strengths and Limitations

A key strength of this study was the use of a smartphone app and accompanying dashboard, which enabled the identification of areas where children were exposed to food advertisements based on their movement patterns. This approach facilitated the identification of areas that would not have been selected by researchers. For instance, children in Södertälje commuted to a shopping center beyond their immediate residential area, illustrating how children often move beyond their local neighborhoods (Multimedia Appendix 2). In Sweden, where public transportation is widely accessible, particularly in urban areas, such mobility among children is common [40]. This behavior-based method captures local context and population characteristics accurately, supporting improved mapping of children’s exposure to food advertising.

In addition, using a smartphone app to collect data is a convenient and cost-effective approach for this target group, as nearly all adolescents in Sweden, and in other high-income countries, own a smartphone that they carry with them throughout the day. The app used in the study allows participants to document their everyday environments in real time, minimizing the need for additional equipment or the presence of researchers. However, the approach also depends on participants’ motivation, engagement, and memory to take pictures, which can vary considerably between individuals. In-app notifications might encourage continued participation and improve compliance throughout the data collection period.

Given the pilot nature of the study, the participant sample was relatively small with 46 children from 4 schools contributing pictures. In 2 schools, hotspot areas were identified from only 5 to 7 participants. Additional (or different) hotspots might have emerged with a larger group of participants. However, in Östermalm, hotspots identified from the first 6 participants closely matched those observed after all 14 contributed, suggesting that small participant groups can still provide meaningful insights.

One limitation of researcher-led mapping of food advertisements was that this study did not account for the type or size of the advertisements. For example, a large billboard promoting a single product likely has a different impact than a small poster outside a grocery store displaying multiple items. Additionally, the cross-sectional design of the study did not capture potential weekly or seasonal variation in advertising exposure.

### Future Research and Implications

In future research, technology-based tools for data collection could be further developed and expanded to provide a more detailed and personalized understanding of the food environment and individual behaviors. For instance, combining outdoor advertising data with information on digital marketing exposure, such as advertisements children encounter on social media, would offer a more comprehensive picture of overall food marketing exposure. This is particularly relevant, given that 86% of Swedish children aged 8‐19 years use social media daily, and studies indicate that they are frequently exposed to food advertisements in these digital environments [41]. Furthermore, the app could be extended beyond food advertising to capture users’ perception of other environmental factors, such as food availability (fast-food restaurants, grocery stores, convenience stores, etc) and accessibility (financial and geographic). The app could also be used to record dietary habits (at a group or individual level), such as meals, snacks, and beverage consumption. Incorporating these additional data types would enable a more holistic assessment of food environments and their influence on individual behavior. Additionally, privacy-preserving features implemented in the app, such as automatic face blurring before transmission, could further improve usability and user trust and boost engagement and long-term use.

In parallel, the dashboard could be adapted accordingly to provide additional heat maps for the new environmental factors and behaviors, as well as additional analytics for examining the associations between food environment and population behaviors (eg, by visualizing clusters of different factors on the same map). Similarly, the dashboard could be further developed to present local evolvement over time, for example, comparing data for weekdays and weekends or tracking changes in food advertisements in any given area throughout the year and across different advertising campaigns (eg, it would be expected that different types of advertisements and offers would be present in periods like Halloween or Easter). Automating the identification and classification of advertisement types using machine learning techniques could potentially improve objectivity, accuracy, and efficiency. Applying analytics at the individual level, instead of aggregated, could enable the potential of the dashboard as a tool for personalized guidance. Finally, future research could investigate whether pictures taken by children could substitute those taken by researchers.

### Conclusions

Using a novel, child-centered mHealth tool combining a smartphone app and a dashboard, this study identified local food advertising hotspots. Within the identified hotspot areas, 77.5% (2291/2955) of food advertisements promoted UPFs, while only 20.8% (616/2955) featured FBVS. Price promotions were found in 23.6% (697/2955) of advertisements, with the majority promoting UPFs. The proportion of UPF advertisements was high, and FBVS low, across all areas, regardless of city size or SES. These findings reveal a food marketing landscape misaligned with dietary guidelines, underscoring the need for policy action and further research.

## Supplementary material

10.2196/70192 Multimedia Appendix 1 List of ultraprocessed foods included in the study.

10.2196/70192 Multimedia Appendix 2 Hotspot maps over Södertälje and Stockholm (Östermalm).

## Abbreviations

FBVS: fruits, berries, vegetables, and seafood
mHealth: mobile health
SES: socioeconomic status
UPF: ultraprocessed food
