# Supplementary material for: Investigating Children’s Exposure to Outdoor Food Marketing in 2 Swedish Cities Using a Smartphone App: Cross-Sectional Study
Source: JMIR Mhealth Uhealth. 2026 Mar 24;14:e70192. doi: 10.2196/70192 (PMC13012817; doi:10.2196/70192)
Supplement: Multimedia Appendix 1 [file mhealth-v14-e70192-s001.docx]

**List Ultra-processed food groups with examples that were considered in the study:**

• Fast food such as pizza, burgers, kebab, hot dogs, nuggets etc

• Sweet snack such as candy, ice cream, pastries, cookies etc

• Salty snacks such as chips

• Ready to eat ready to heat meals such as instant noodles, pies, frozen dishes

• Processed meats such as sausages, ham, bacon

• Sweetened dairy products

• Sweetened breakfast cereals and cereal bars

• Pre-packaged sauces, condiments and dressings

• Pre-packaged breads such as those with high sugar content

• Sugary and artificially sweetened beverages

• Alcoholic beverages

* Several categories include exceptions. For example: not all frozen meals meet the criteria for being ultra-processed.
