# Supplementary figures and images for: Investigating Children’s Exposure to Outdoor Food Marketing in 2 Swedish Cities Using a Smartphone App: Cross-Sectional Study
Source: JMIR Mhealth Uhealth. 2026 Mar 24;14:e70192. doi: 10.2196/70192 (PMC13012817; doi:10.2196/70192)

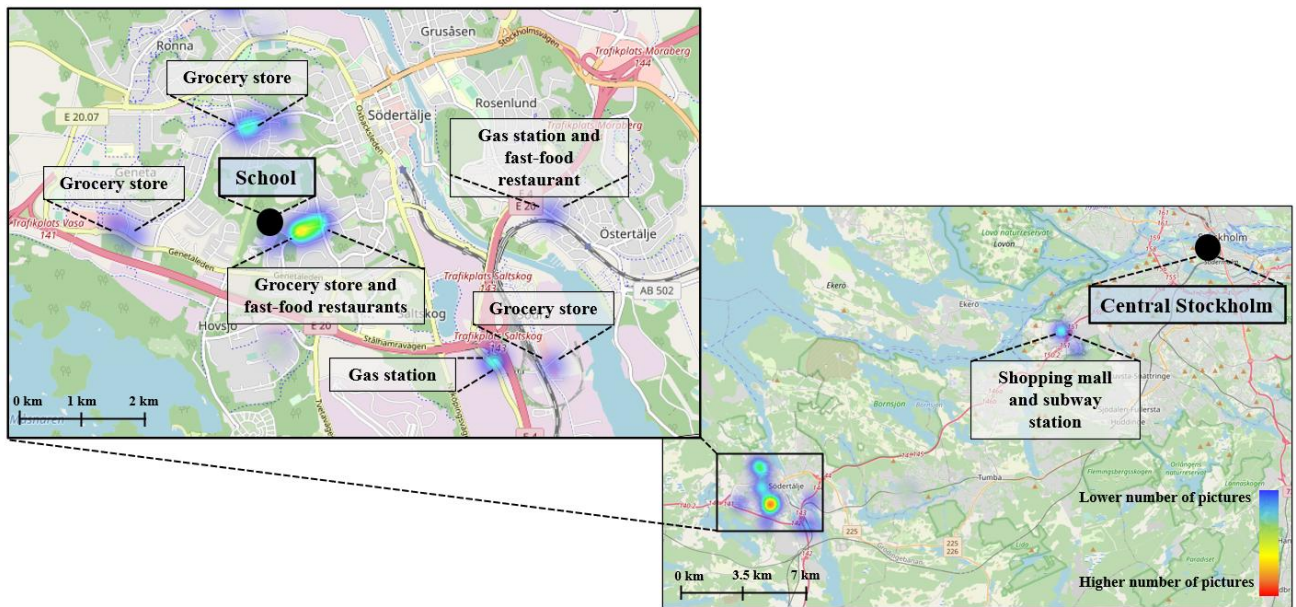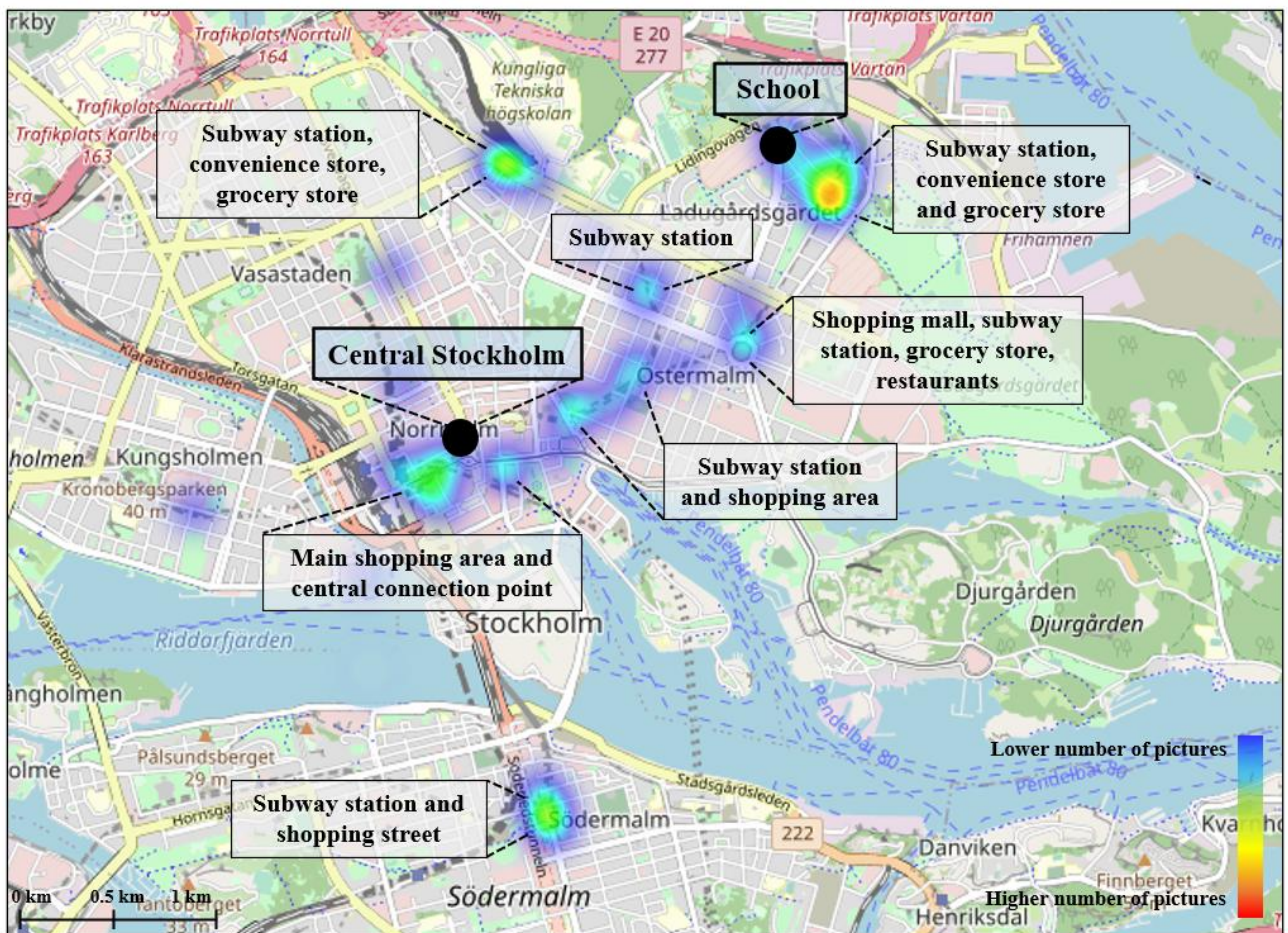

Supplement: Multimedia Appendix 2 [file mhealth-v14-e70192-s002.pdf]
